# Implementing a clinical cutting-edge and decision-making activity: an ethnographic teamwork approach to a molecular tumorboard

**DOI:** 10.1186/s12913-020-05786-2

**Published:** 2020-10-07

**Authors:** Nathalie Bot, Mathias Waelli

**Affiliations:** 1 Institute of Global Health, University of Geneva, Geneva, Switzerland; 2 EHESP, French School of Public Health, EA7348 MOS, Rennes, France

**Keywords:** Molecular tumorboard, Precision oncology, Learning organization, Interdisciplinary team, Care customization

## Abstract

**Background:**

New technology implementation in healthcare must address important challenges such as interdisciplinary approaches. In oncology, molecular tumorboard (MTB) settings require biomedical researchers and clinical practitioners to collaborate and work together. While acknowledging that MTBs have been primarily investigated from a clinical rather than an organizational perspective, this article analyzes team processes and dynamics in a newly implemented MTB.

**Methods:**

A systemic case study of a newly implemented MTB in a Swiss teaching hospital was conducted between July 2017 and February 2018, with in situ work observations, six exploratory interviews and six semi-structured interviews.

**Results:**

An MTB workflow is progressively stabilized in four steps: 1) patient case submissions, 2) molecular analyses and results validation, 3) co-elaboration of therapeutic proposals, and 4) reporting during formal MTB sessions. The elaboration of a therapeutic proposal requires a framework for discussion that departs from the formality of institutional relationships, which was gradually incepted in this MTB.

**Conclusions:**

Firstly, our research showed that an MTB organizational process requires the five teaming components that characterizes a learning organization. It showed that at the organizational level, procedures can be stabilized without limiting practice flexibility. Secondly, this research highlighted the importance of non-clinical outcomes from an MTB, e.g. an important support network for the oncologist community.

## Background

In healthcare, in particular the field of precision oncology, new technologies are redesigning work organizations. The advent of targeted therapies, including the identification of genetic mutations active in cancer, i.e. driver mutations, and the development of affordable high throughput sequencing technologies, has opened up the field of precision oncology [1–3]. In developing tumor molecular profiling, the mission of precision oncology is to detect so-called actionable genomic alterations, or actionable variants [4], that can be specifically targeted by existing therapies, i.e. targeted therapies or immunotherapies, or therapies in clinical development.

In this respect, sequencing platforms in university hospitals have gradually resorted to predictive oncology approaches, and organizing their activities around multidisciplinary Molecular Tumorboards (MTB) [5]. Unlike conventional tumorboards, which are organized into organs/systems, MTBs analyze genomic profiles from tumors in patients with limited care options. What is key to these MTBs is not the coordination of care as in conventional tumorboards, but the interpretation of tumor molecular profiles that underpin treatment recommendations.

Our literature review showed that MTBs were diverse, ranging from small advisory boards providing treatment recommendations, to boards incorporated into large precision oncology-based clinical trials; all differed in composition, expert categories, genomic areas, bioinformatics techniques and workflows [6–21].

Despite this diversity, the implementation of any MTB in supporting such biomedical interpretative processes, from sequencing data interpretation to hypothesizing therapeutic proposals, requires a bioinformatics, molecular, genetics and clinical analytical capacity and expertise. Consequently, experts enrolled in any MTB must comprise clinicians, pathologists, bioinformaticians, biologists and geneticists; all participating in inter-professional teamwork.

The second feature of any MTB is the ability to understand the rapid evolution of technologies, (e.g. genetic panels, analytical software, knowledge libraries or clinical databases) and biomedical knowledge, as well as continuous therapy development. These aspects alone represent particular challenges for establishing standardized protocols and guidelines that inform the activities of these tumorboards.

Publications that share experiences of MTB implementation, very often report their data after one or 2 years of activity; i.e. MTB decisions and activity processes are presented in the form of patient flow diagrams or decision trees, detailing case evaluation work [6, 22], which mostly focus on clinical outcomes. These reports yield relevant data, particularly on MTBs generating therapeutic proposals. However, how MTB members are organized on a daily basis, to collectively produce analyses and clinical recommendations remains insufficiently documented. It is not clear how inter-professional teamwork is organized, and what processes underlie activities within these MTBs, e.g. how do MTBs integrate results from cutting-edge research? Thus, important elements are missing from the literature on interdisciplinary working practices in these environments. Based on an ethnographic approach, the objective of our study was to analyze organizational work processes inside a recently implemented MTB.

## Methods

### The study setting

The documentary research was conducted in two phases. We searched for institutional documentation as well as articles from the local press (both specialist and general), to determine the extent of the network and identify key establishing players. For each of these actors, we performed a review of their work to; 1) better understand the scientific issues contributing to network dynamics, and 2) to conduct interviews. Six exploratory interviews with key network players were conducted. The interviews lasted between 30 and 60 min and were not recorded, but notes were taken. During interviews, key players repeatedly referred to a newly implemented Molecular Tumorboard (MTB), as an innovative facility for precision medicine, and a relevant empirical object for our investigations. Accordingly, local and professional journals (citations withheld to preserve MTB anonymity) represented this MTB as an access point to medical innovation and cutting-edge clinical decision-making (using genomics and bioinformatics) for patients and their treating physicians.

### Systemic MTB case study

To better understand the teaming process of an MTB, and its impact on clinical decision making and innovation integration, we adopted a systemic qualitative methodology based on case studies in the social sciences [23]. With a case study approach, the investigation focused on the local context of work. This allowed us to investigate the perspectives of the different actors involved in the process, and focus on their workflow dynamics. This approach required two types of data collection, in addition to the documentary research and exploratory interviews, these were in situ observations and semi-directive interviews.

### In situ observations

For 6 weeks, between January 17th and February 28th, 2018, the principal investigator (PI) (NB) attended all weekly meetings at the MTB. There were two types of weekly meeting: 1) an MTB session where team members and referring oncologists presented and discussed therapeutic proposals, and a 2) “preparatory meeting”, restricted to MTB team members only. During this latter meeting, the team discussed genomic findings and clinical data for patients.

Between the study dates, the PI observed five preparatory meetings and four MTB sessions. At each session, the PI recorded elements such as location, duration, purpose of exchanges, the number of people and the materials available (circulating documents). For preparatory meetings, attention was focused on the interpretation of genetic results. While the PI profile (molecular biology) facilitated subject familiarization, a search for extra information was also performed. In addition to organizational and technical observations, the PI noted elements related to questions from MTB participants regarding uncertainty on the interpretation of molecular profiles or clinical images.

During MTB sessions, the PI’s attention was focused on interactions between referring physicians and the MTB team, and concerns expressed by physicians.

Finally, study observations were not extended to collaborative work in the pathology laboratory, where the first part of validation analyses usually occurred. The absence of observations in this area highlights the limitations of our study, which lie in its inductive approach.

### Semi-structured interviews

Following the MTB observational period, in-depth semi-structured interviews (*N* = 6) were conducted with a representative, preferably permanent member of each discipline, of the MTB team; an oncologist (ON), a pathologist (PA), a bioinformatician (BIO), two oncogeneticists (OG), and one research assistant (RA). They were interviewed individually at their workplace (office) for 60–90 min. Each interview was recorded, fully transcribed, and followed an interview grid, comprising three major discussion elements corresponding to study objectives: 1) the history behind implementation of the MTB, 2) collaborative working modalities, and 3) modalities of genomic information interpretation. In addition to establishing an interview grid that was developed for this study (see Additional file 1), interview pre-preparations consisted of reading interviewees’ main scientific articles, searching biographies on the institution website, and accessing articles or the local press relating to media interventions by interviewees.

### Analyses

Data were subjected to a triangulation process where interview analyses made it possible to identify themes. These themes were put into perspective with data from observations and documentation. No coding rules were pre-established. Themes were built in an abductive and dynamic way.

More precisely, we sorted data from interviews that allowed us to reconstruct the different stages of MTB workflows. Thus, we captured the scheduling of tasks, and complemented the fragmented vision of the process from in situ observations. We then compared our workflow reconstruction with other MTBs from the literature [3, 5, 6, 12, 15].

Subsequently, we then identified implementation issues perceived by the various respondents, according to the place they occupied in the division of tasks. We also identified a number of concerns shared by all team members. We compared our results with our observation notes, and articles dealing with precision oncology implementation issues [18, 23].

These exercises confirmed that issues highlighted in our research corresponded to current issues in the field.

## Results

The main challenges underlying the cutting-edge clinical decision-making process, is the construction of off-label scenarios for patients, where beneficial treatment actions for unusual cancers and/or genomic alterations are identified and reasonably predicted. This model results from a long process; presented in two parts; the first describes four workflow organizational steps, starting with the referring oncologist request, through to the therapeutic proposal presented at MTB sessions.

The second part highlights how three specific issues were addressed by MTB members: 1) researching the scientific literature for the best therapeutic options, 2) assessing patient benefits, and 3) establishing work practices. Each factor highlights the teams’ challenge in collectively responding to the MTB’s mission of delivering genetically informed guidance.

### PART I: the workflow

**Fig. 1:**
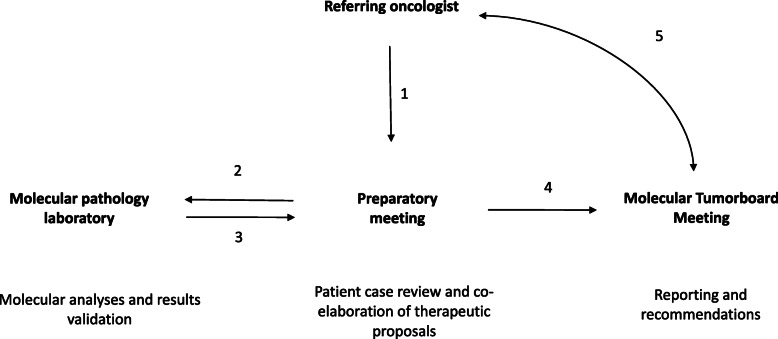
Overview of the MTB workflow. Each patient case was discussed (1) during the preparatory meeting. The molecular pathology team performed genetic analyses and clinical annotations according to MTB recommendations (2). A second discussion was conducted after the results were analyzed (3), but within the framework of the preparatory meeting. Finally, a therapeutic proposal was finalized (4), and reported back to the treating oncologist at the MTB session (5)

At the time interviews were conducted, the MTB had been in situ for almost a year, processing approximately 7–8 patient cases per week. During the year following its launch, MTB organization had gradually modified to adapt to the growing demands of patients cases requiring MTB expertise. Board activity had settled into a routine that we reconstituted by cross-checking interviews with observational data. We categorized activity workflow into four organizational steps (Fig. 1): 1) patient case submissions, 2) molecular analyses and results validation, 3) co-elaborating therapeutic proposals, and 4) reporting during formal MTB sessions.

#### Patient case submissions: a space for discussion

During the case submission phase, the process was both structured and determined by an informal framework that facilitated exchanges between actors.

Our data showed that the treating oncologists initiated a consultation with the MTB. Their request came when a patient had progressed onto several lines of standard therapy, or for a patient with refractory metastatic cancer. In this MTB, the most common conditions were: “*pathological conditions for which we know that patients do not necessarily respond to treatments...mainly pancreatic cancers, colon cancers, breast cancers, essentially the same pathological conditions return*” (RA). During our study, we observed that attending physician requests were made informally by email or simply via discussion with an MTB team member.

Once a request was made, the MTB RA collected the patient’s signed informed consent sheet. If the patient was treated outside the hospital, i.e. in the private sector, the RA sent a request to the relevant institution to have samples (biopsy and blood) forwarded to the molecular pathology laboratory of the University hospital, where additional histopathological and genomic analyses were performed, as recommended by the MTB team. Each new patient submission was initially discussed at the weekly interdisciplinary “preparatory meeting”. This meeting occurred in a small classroom in the pathology department, and gathered a restricted body of experts representing four major disciplines: oncology, pathology, oncogenetics and bioinformatics. A few hours before the meeting, the RA sent out information including new submissions and those undergoing genomic analyses and/or ready to be presented at the next MTB session (which occurred a few days later).

Thus, at the preparatory meeting, participants directly consulted patient files via the electronic health record (EHR) system, and if necessary, printed details for the preparatory meeting. During the meeting, new submissions, approximately 7–8 per week (as observed during our study), were reviewed and discussed by 7–10 attendees. Sequencing data interpretation of ‘in progress cases’ was the most time-consuming and contentious element of the meeting. The work dynamic underlying collective case assessments was conditioned by two biological and clinical priorities: urgency and access to biological material. In emergencies, patients with rapidly progressing disease were prioritized. As for biological material (biopsy), it was important to have sufficient quantities to ensure sequencing success, because “*If we are given 10% of tumor cells to view on a slide, we can also try to sequence the material, but without any guarantee of finding anything. With only 10% of tumor cells, a heterozygous mutation would be at 5%, and 5% is the detection limit for sequencing*” (BIO). Consequently, when the biological material represented approximately 70–80% of the tumor, detecting genomic alterations was stronger. However, at ≤30%, the absence of genomic alterations did not mean there were none, simply that not enough material was present for definitive analysis.

#### Molecular analyses and results validation: an interdisciplinary collaboration

Results analyses and validation often went back and forth (depending on case complexity) between teams, pathology teams and MTB members.

After histopathological biopsy analysis and cell selection for sequencing, sequencing analysis was performed in two steps. The first step assessed the validity of the sequencing results, while the second determined its functional and potential clinical significance. Questions underlying this analytical process were summarized by an expert: “*When you have a mutation, you wonder what is its functionality, what is its pathogenicity? But the first question we ask ourselves, is it a sequencing error?* “(BIO).

Data analyses were complex and depended on who performed them. This was why data interpretation underwent peer review by laboratory members. For example, Next Generation Sequencing (NGS) 400 panel results were validated by two laboratory members: the data was first viewed by a bioinformatician or biologist, who determined amongst the genomic alterations, which ones were real and which were artifacts, then he/she analyzed variants and sorted them according to pathogenicity. A second person, a biologist or pathologist then processed these data to validate them.

This analytical process included further clinical annotations. Laboratory pathologists and biologists reviewed the scientific literature and used relevant databases listing pathogenic variants [24–26]. These steps were recorded and documented in an internal laboratory sequencing database which, in addition to constituting the central database of the laboratory, included a notification system which automatically informed an individual working on the analysis of any changes made to them. This feature improved sequencing annotation quality and reduced error rates.

The number of variants identified in each sequencing sample varied greatly, depending on the quality and quantity of tumor cells in the sample, but also on tumor type. According to one expert, the NGS400 panel detected small but variable numbers of significant mutations: “*There may be zero, there may be sixty, there may be three...but generally speaking, we don’t have many, at most about ten...*”(BIO). In general, approximately ten mutations were identified, of which 5–8 were selected by the laboratory team. Information on pathogenic variants were integrated into the laboratory’s internal database, and into integrative reports made available to MTB members via electronic records.

#### Co-elaborating therapeutic proposals

The elaboration of a therapeutic proposal required a framework for discussion that departed the formality of conventional institutional relations, and was gradually incepted by the MTB.

Data from genetic tests, validated by the pathology laboratory and made available to MTB experts as an integrative report, were discussed at preparatory meetings. Between new requests and those in progress, the team reviewed approximately 15 cases per hour. Discussions on sequencing results occupied most of the meeting, and was the reason why this preparatory meeting was established. Although this session format was not planned at the MTB’s inception, this preparatory meeting, which helped prepare the MTB session, proved invaluable. Its weekly format included informal discussions, interpretation of newly received data, formulating questions and making decisions. Importantly, sequencing results were examined in a broader context, accounting for a patient’s clinical history. Test results, as a list of variants, were reviewed by the group and if genetic findings were considered sufficiently informative, they were presented at the next MTB session.

A final step in this process was the transformation of biological data into therapeutic proposals. This step, carried out by an oncologist, consisted of researching the literature and databases to formulate proposals based on the therapeutic resources available. The description of the treatment process showed that all decisions were based on clinical expertise and interpretation of the scientific literature. Thus, from the 5–8 genetic variants selected by the pathology laboratory, then examined by the MTB team, the oncologist focused the analysis on one or two variants per patient case. To formulate each therapeutic proposal, the oncologist consistently combined information from evidence-based forums: e.g. a known and well-studied biological mechanism, a well characterized and effective drug, and complementing valid models from phase I or II studies. These results were presented to the referring physician during the next MTB session. The referring oncologist, who knew the patient, evaluated the feasibility of the proposed treatment.

#### Reporting during formal MTB sessions: results reporting and implementation issues

The MTB plenary meeting was a formal space for reporting decisions taken during preparatory meetings, at biological and clinical levels. The forum facilitated the exchange of views with other actors to evaluate recommended treatments and implementation issues.

The weekly MTB session provided genomic result summaries and treatment recommendations to attending physicians. This was organized in tandem with a team from another university hospital, and occurred in a video-conferencing format. The attending physicians were invited to join the online meeting, at the point where their patient was discussed. In the room, the MTB team gathered around a V-shaped table. On each side of the table, interns listened and presented their own patients for review. At the beginning of the presentation, each attending physician presented a brief oncological history of their patient, then the MTB team presented a summary of the genetic data and the therapeutic proposals, followed by a referenced inventory of supporting evidence. The MTB panel could either recommend standard or off-label treatments, and/or referral to a clinical study. In some cases, no treatments were recommended, or the panel advised against a treatment plan. Finally, family anamnesis was recommended by the oncogeneticist, when a familial genetic predisposition to cancer was suspected.

We observed that in comparison with preparatory sessions, the presence of the attending physician at MTB sessions, influenced exchanges: e.g. the MTB team could directly ask the referring oncologist whether additional analyses or a new biopsy was possible. If clinical trials were available, the attending physician could inform the team about the patient’s current health condition and eligibility for clinical trial enrollment. We noted that when proposed treatments were non-standard, the attending physician tended to worry about the procedures, from an insurance company viewpoint. However, there was little discussion regarding genetic findings between attending physicians and MTB teams, probably because of a lack of a common knowledge base. As one MTB member noted*,“... there is not much discussion, because only they (the MTB team members) know about it. When everyone knows about it, it is classic care”* (RA). Finally, during this meeting, information on ongoing clinical trials or any additional analyses were shared between the two MTB teams. A report summarizing the results and recommendations was then assembled (by the oncologist and the RA), and sent to each attending physician. It was also integrated into the patient’s electronic record.

In summary, the turnaround time between case submission and final recommendation was 2–3 weeks. Each patient was discussed during the preparatory meeting. The molecular pathology team performed genetic analyses according to MTB recommendations, and a second discussion, still within the framework of the preparatory meeting, occurred. Therapeutic proposals were developed on this basis, and results were reported to the treating oncologist during the MTB session.

### Part ii

In a context where knowledge is often not supported by scientific research, MTB experts set up working modalities, allowing for the elaboration of therapeutic strategies, but mindful of different constraints e.g. technical, clinical, economical and ethical. Three factors were significant to this thinking.

#### Integrating cutting-edge research data into clinical decisions: browsing the grey zone

Validation work was initiated in the molecular pathology laboratory which controlled the quality of genetic results, validated genomic alteration analyses, and proposed a list of variants, classified according to pathogenicity. Thus, 5–8 variants were selected by the laboratory and discussed by the MTB team, allowing the MTB oncologists to formulate therapeutic proposals. A thorough selection allowed the oncologist to focus on one or two variants at most. Accordingly, the oncologists’ analyses were based on the scientific literature and their own expertise. First, actionable variant sorting was performed by consulting large comprehensive databases [24–26], and digesting clinical and genomic research articles [27]. For the clinician, this work was enormous “*you have to read a lot of literature, you have to quickly read and interpret articles and clinical studies*” (ON). Thus, there was a requirement to prioritize variants, which was summarized as follows: for each genomic alteration, it was important to obtain a coherent combination of information at several levels, i.e. a known and well-studied biological mechanism, a well characterized drug that was effective and accessible, and complementing valid models from phase I or II clinical studies. The clinician explained: “*If you have a well-defined signaling pathway, a well characterized drug, very convincing in vitro or mouse models, to which you add a phase I or II study, or a small study involving fifteen patients, you are more convinced when compared to a completely new drug with results that cannot be linked to current understandings of cancer biology. It reassures us to have coherent proposals at all levels*” (ON). In other words, the therapeutic proposal was intended to make the most of published clinical and pharmacological data. During scientific literature research, the clinician was most often confronted with small studies of modest size, of modest quality, and rare situation case reports. The oncologist also described their subject area as being on the border of what was known and unknown: “*We have to cover the grey zone, which ultimately covers quite a lot of diseases in good quality publications, for example phase II studies, with fairly good, if not very interesting results. Or very small studies on extremely rare diseases that do not have the same level of evidence as very large studies and official recommendations*” (ON). To make a personalized treatment proposal, the oncologist used a systemic approach based on consistency between several published outcomes at different evidence levels (clinical, preclinical, biological and pharmacological), while navigating areas of uncertainty. In essence, in this innovative and cutting-edge context, MTB experts had to compensate for the absence of unequivocal, proven results, by cross-referencing information from the literature with their own professional experiences.

#### Assessing patient benefits during preparatory meetings

Each MTB activity depended on a combination of several contextual factors such as organizational, political, social, or financial issues that eventually determined therapeutic choices for patient benefit. For a patient to benefit from MTB analyses, several obstacles had to be overcome. In addition to adequate tissue for sequencing, the patient had to be in good general condition to undergo treatments, often beyond standard requirements. Even if this patient met these criteria, and the tumor genomic profile was sequenced and validated, the clinical utility of identifying molecular alterations was still somewhat limited [5]. With regards to the MTB, the following feedback reiterated this view: “*There is one patient for whom we had feedback, we performed sequencing and found something interesting, he was really close to the end of his life, we treated, it worked, he gained six months and then the tumor became* resistant and it was unfortunately over” (PA). A possible reason for this poor outcome was not due to a lack of treatment proposals by the MTB, but rather due to evolving drug resistance, possibly do to an emerging molecular sub-clone [28].

Another reason in poor outcomes is difficulties in accessing treatments [9]. Indeed, the MTB’s recommendations were conditioned by available therapeutics: “*we can perform the best tests in the world, have the best specialists around, but we dependent on the pharmacological means at our disposal*” (ON). An oncologist from the MTB determinedly explained, that while searching for appropriate drugs, only those available in Switzerland were prioritized because “*telling a patient there may be a drug that perhaps works, but it is in New York, is not ideal. So this has to be taken into account, because it remains a clinical activity. We need to be aware of studies and medications. The patient is not served if he does not have access to medication that’s is offered to him*” (ON). In addition, when a clinical trial was within a patient’s reach, the patient often had to meet very strict inclusion criteria.

A second impact on access to patient treatment related to the format and mission of the MTB. This MTB welcomed and reviewed all submitted patient cases, as long as the two criteria, biopsy availability and good condition were met: “*Pragmatically, the priority is to help patients, the specific patient who has been referred to us. This priority remains our primary objective. The tumorboard is therefore not a research activity but a clinical activity*” (OG). Interviewees stressed that each request deserved to be examined because one of the assets of the MTB approach was its predictive value: it was not only a question of proposing new therapeutic options, but also identifying those tumor resistant drugs, thereby preventing the patient undergoing unnecessary treatments. As one expert added: “*Today in oncology, the most difficult thing say to a patient is, now we have reached the end of treatment prospects for you. We have to support you and take care of you, we will continue to do it in a different way, but we have to stop. Everything that continues from here will not benefit your quality of life.*” (OG). The MTB collective decision-making process assigned a weight to several decision factors such as patient health conditions, tissue sample availability or drug accessibility.

#### Setting up work practices

Clinical decision-making processes were subject to ever evolving technical, academic and clinical environments. This element was often challenging, as pointed out by members when initially implementing the MTB. Interviewees stressed the slowness of process implementation. They were aware of a collective learning process, and noted the almost confidential nature of the first MTB meetings, and the associated feelings of inexperience. As one interviewee recounted: “*Things finally came together, without necessarily thinking about everything in advance. And then it started very slowly. At first there was maybe one case, and three people. We didn’t want it to start quickly, because we were engaging in something that none of us really knew anything about”* (OG). Notably, the preparatory meeting for the MTB was later established after one of the members remarked: “*We cannot arrive at the MTB session, each of us having prepared our own input...as if we were looking at patients, each with our own minor perspective.... I said: We have to have another meeting: the preparatory meeting*” (OG), thus establishing the requirement for an inter-professional collaboration to resolve ambiguous or complex issues, by integrating and discussing broader clinical contexts for each genetic finding. Interestingly, pathology laboratory members were also included in these preparatory meetings, shortly after the MTB was set up, to provide in-depth technical details on genetics data.

A second adjustment in work practices involved accepting only patient cases who were sufficiently fit. One oncologist recalled: “*it was still a bit early, because we all had to learn. We saw cases, very old people presented to the molecular tumorboard, and by the time the evaluation was completed, they died from another cause. This example is extreme, but typically there are people for whom the molecular tumorboard is out of place* (OG).”

At the time interviews were conducted, the MTB had been in place for 1 year, processing 7–8 new cases per week. In this context, even though a routine was in place, the desire to rely on comprehensive guidelines was stressed during interviews. One member commented: *“Currently, the guidelines do not exist. You don’t have a document that states for all molecular tumorboards what must be done. It’s true that at the moment, it’s complicated because the platforms are different, not everyone uses the same tools*” (OG). Thus, there were no accepted guidelines determining, for example, the criteria for patient submission to MTB expertise, the most appropriate panels and technologies, the level of evidence required for actionable variants, or workflow organization or tumorboard composition [29].

## Discussion

Our study, focusing on a newly implemented MTB, showed that its implementation was subjected to an evolutionary process. As a result, MTB members learnt to operate flexible organization based on standardized (or stabilized) sequences of practices (each located in dedicated spaces; the laboratory for bioinformatics and pathological analyses, the preparatory meeting classroom for collective assessments, and the tumorboard videoconference room for case presentations and final recommendations), as well as developing competences to adapt to specific needs and constraints of patient cases.

Firstly, our results showed that MTB organizational implementation steps met “Teaming” principles, and its five components as proposed by Edmondson [30]: i.e. framing, psychological safety, boundary spanning, leadership and organizational learning.

The “framing”, component consisted of a shared vision and objective by all team members. Here, the common MTB objective was centered on patients, and focused on providing a service to the attending oncologist, contrarily to the aforementioned genome based cohort studies. This MTB mission was well understood and supported by all MTB members. MTB clinicians often mentioned the importance of integrating bioinformaticians and pathologists into clinical decision-making processes, to ensure shared objectives.

The “psychological safety” component, enabled an environment for free self-expression within a team, and was achieved by setting up informal, preparatory meetings. In this context, cross-examining perspectives (e.g. genetics, clinical, pathological, bioinformatics and pharmacological) during preparatory meetings were essential factors when a patient’s health was at stake.

“Boundary spanning” referred to the team’s ability to go beyond the boundaries of status or level of knowledge [31]. It constituted a key part of the examination process during these preparatory meetings. The fact that laboratory team members were included in preparatory meetings so soon after the MTB was set up, constituted an important “boundary spanning” step. It created interactions between health professionals and researchers, generating common goals.

Leadership is an essential component of the Teaming principle. Our observations showed that the distribution of leadership within the MTB team generally depended on the type of decision and the specific expertise of each player, however decisions were collegial. Decisions systematically evolved from exchanges between MTB members.

A last component of the Teaming process concerned “organizational learning”, which was transversal, as any changes implemented in the tumorboard’s organization were characteristic of the concept. In other words, the work organization established up by the MTB could stabilize, but responses, to specific situations were required to change constantly. These changes in work practices were typical of a “learning organization”, and reflected the ability to account for each situation, thereby improving organizational effectiveness. Paradoxically, the MTB members who participated in our study, like those involved in other MTBs in the literature, advocated a global standardization of responses and procedures at all levels [7, 8, 29, 32], highlighting a tension between procedure stabilization and learning dynamics. Edmonson’s framework showed us, that at an organizational level, procedures can be stabilized without limiting practice flexibility [30].

This phenomenon was related to the emerging theoretical framework of “care customization” [33] inspired by general managerial theories [34], but adapted to the healthcare arena [33]. This approach showed that attending to the individual need of each patient or professional, required a combination of standardized organizational steps, including the development of social competencies for healthcare workers. Thus, the MTB was a good example of how” care customization” was implemented, by combining the positive effects of standardized processes of interdisciplinary work, to elaborate innovative and adapted therapeutic proposals.

Secondly, at first sight, the evolution of work practices and MTB team performances appeared partly disconnected from a patient’s clinical outcomes. Interestingly, during our observations, patient follow-up by the MTB team was not yet fully implemented. However, in this instance we have an example of a health service centered on patient quality of life, by investigating treatment options, and identifying ineffective intended treatments. Importantly, the MTB also encouraged exchanges between hospital professionals and referring oncologists, thus offering a service to community-based practitioners. How and why this service could be used, and its impact on the community may open up new avenues of research in the future.

This case study had a number of limitations. It involved a single tumorboard in a limited space, and lacked the ability to assess MTB clinical outcomes. However, our approach highlighted the importance of interdisciplinary dynamics in a learning organization such as an MTB. It also underlined the importance of organizational dimensions, which made it possible to articulate the need to stabilize work processes, while remaining flexible in terms of responses offered to patients and other healthcare professionals.

## Conclusions

Within the context of promoting patient-centered approaches [35–37] organizations must work towards greater flexibility, and therefore rely on multidisciplinary approaches. These transformations require adjustments by health professionals in developing new ways to work together, for which there are few guidelines. Thus, the implementation of an MTB, often characterized by a strong level of personalization, could serve as an effective “laboratory” for research on interdisciplinary work approaches, and a template for all health organizations.

## Supplementary information

**Additional file 1.** Interview questionnaire.

## Data Availability

Datasets from this study are available from the corresponding author on request.

## Abbreviation

MTB: Molecular tumorboard
