# Supplementary material for: Implementing a clinical cutting-edge and decision-making activity: an ethnographic teamwork approach to a molecular tumorboard
Source: BMC Health Serv Res. 2020 Oct 7;20:922. doi: 10.1186/s12913-020-05786-2 (PMC7542871; doi:10.1186/s12913-020-05786-2)
Supplement: Supplementary file 1 — Additional file 1. Interview questionnaire. [file 12913_2020_5786_MOESM1_ESM.docx]

**Additional file 1 : Interview questionnaire**

## the history behind implementation of the MTB

1. Do you know when was the MTB implemented and according to what needs?
2. Were you involved in setting up this MTB? And if so, what was your role?
3. What is the level of collaboration with the MTB at the other university hospital?
4. Apart from this collaboration, do you collaborate with other MTBs in Switzerland or other Institutions and if so, what is the extent of this collaboration?
5. In your opinion, what are the challenges to make this system - Molecular Medicine- evolve in the coming years?

## collaborative working modalities

1. When did you start working in this MTB and how did you get into it?
2. Can you describe the sequence of tasks performed each week between the preparatory meeting (on Wednesday) and the MTB session on the following Friday?
3. Can you specify in this sequence, what are your tasks- and the time they take- and those of your collaborators?
4. Are there any experts with whom you already collaborated before the MTB was implemented?
5. Since you started working with the MTB, what elements in its organization, or in your tasks, have changed?
6. Have you already participated in or attended other molecular tumorboards and if so, what are the similarities or differencies in organizational elements?
7. What elements would now need to change in order to develop the MTB in the coming years? What are the challenges?

## modalities of genomic information interpretation

1. What is in general, the profile of patient cases (type of cancer, age, or other) analyzed by the MTB ? Which patients can benefit from this MTB ?
2. Do you have criteria for refusal/acceptance of patient cases ? If so, what are they?
3. What are the recurring problems when interpreting the data?
4. How do you prioritize the identified mutations, if there are several? How do you sort the data?
5. Why did you choose these panels and these lab tests in particular to perform genomic analyses?
6. What databases do you use and why ?
7. What other criteria should be considered when making therapeutic proposals?
8. Do you know if MTB recommendations are being followed and can you estimate their clinical efficacy?
9. Are any of your results used in your research?
